# Does diet quality matter? A secondary analysis of a randomized clinical trial

**DOI:** 10.1038/s41430-023-01371-y

**Published:** 2023-11-28

**Authors:** Hana Kahleova, Haley Brennan, Tatiana Znayenko-Miller, Richard Holubkov, Neal D. Barnard

**Affiliations:** 1 Physicians Committee for Responsible Medicine, Washington, DC USA; 2 School of Medicine, University of Utah, Salt Lake City, UT USA; 3 Adjunct faculty, George Washington University School of Medicine and Health Sciences, Washington, DC USA

**Keywords:** Nutrition, Obesity

## Abstract

This secondary analysis assessed the association of a plant-based index (PDI), healthful (hPDI), and unhealthful (uPDI), with weight loss in overweight adults. Participants (*n* = 244) were randomly assigned to a vegan (*n* = 122) or control group (*n* = 122) for 16 weeks. Three-day dietary records were analyzed and PDI indices were calculated. A repeated measure ANOVA was used for statistical analysis. All three scores increased in the vegan group; the effect sizes were: PDI +10.6 (95% CI +8.6 to +12.6; *p* < 0.001); hPDI +10.9 (95% CI +8.4 to +13.4; *p* < 0.001); and uPDI +5.4 (95% CI +3.4 to +7.4; *p* < 0.001). The change in all three scores significantly correlated with change in body weight: PDI (r = −0.40; *p* < 0.001); hPDI (r = −0.37; *p* < 0.001); and uPDI (r = −0.21; *p* = 0.002). These findings suggest that minimizing the consumption of animal products and oil may be an effective weight loss strategy in overweight adults. ClinicalTrials.gov number, NCT02939638.

## Introduction

Plant-based diets have been associated with lower body weight and reduced type 2 diabetes prevalence. Based on observational studies, some have proposed quantifying the healthfulness of plant-food dietary patterns, creating plant-based (PDI), healthful plant-based (hPDI), and unhealthful (uPDI) plant-based dietary indices [1]. However, the association of such categorization with body weight has not been confirmed in randomized trials. A secondary analysis of previously published data [2] assessed the association of PDI, hPDI, and uPDI in the context of a vegan diet, with weight loss in overweight adults.

## Methods

As described previously [2], this randomized trial was conducted between January 2017 and February 2019 in Washington, DC. This study follows the Consolidated Standards of Reporting Trials (CONSORT) reporting guideline. The protocol (Supplement 1) was approved by the Chesapeake Institutional Review Board. All participants provided written informed consent.

Participants were randomly assigned to a vegan or control group (Suppl. Fig. 1). The vegan group was asked to follow a low-fat vegan diet consisting of fruits, vegetables, grains, and legumes. The control group was asked to make no diet changes. No instructions on diet quality were given to either group.

At baseline and week 16, a 3-day dietary record (two weekdays and one weekend day) was completed by each participant and analyzed by a registered dietitian certified in the Nutrition Data System for Research [3]. The PDI, hPDI, and uPDI were calculated [1]. A repeated measures ANOVA was used by a statistician blinded to dietary interventions. Results are presented as means with 95% confidence intervals. Pearson correlation was used to evaluate the magnitude and significance of the association between the changes in body weight and changes in all three indices and their individual food components. After Bonferroni correction, *p* values less than 0.003 (0.05/17) were considered significant. In addition, a stepwise forward multiple regression analysis was fit (using all food components as candidate predictors) to determine food components independently predictive of change in body weight.

## Results

Of 3115 people screened by telephone, 244 overweight adults met participation criteria and were assigned to the vegan (*n* = 122) or control (*n* = 122) groups. The analysis included 223 (91.0%) completers.

### Dietary intake

Self-reported energy intake was reduced in both groups, more in the vegan group (effect size: −367.6 kcal/day [95% CI −536.8 to −198.5]; *p* < 0.001). The macronutrient content did not change significantly in the control group, while the vegan group participants increased their intake of carbohydrate (effect size: +22.5% of daily energy [95% +20.1 to +24.9]; *p* < 0.001) and fiber (effect size: +11.5 g/day [95% +8.1 to +14.8]; *p* < 0.001), and reduced consumption of fat (effect size: −18.0 % of daily energy [95% −20.1 to −15.8]; *p* < 0.001), protein (effect size: −4.7 % of daily energy [95% −6.1 to −3.4]; *p* < 0.001), and cholesterol (effect size: −215 mg/day [95% −261 to −169]; *p* < 0.001).

### Body weight and body composition

Body weight decreased in the vegan group (effect size: −5.9 kg [95% CI −6.7 to −5.0]; *p* < 0.001). The majority of the weight reduction was due to the reduction in fat mass (effect size: −4.1 kg [95% CI −4.7 to −3.5] kg; *p* < 0.001), and visceral fat (−209 cm^3^ [95% CI −304 to −114]; *p* < 0.001).

### PDI, hPDI, uPDI

All three scores increased in the vegan group, compared with no change in the control group; the effect sizes were: PDI + 10.6 (95% CI +8.6 to +12.6; *p* < 0.001); hPDI +10.9 (95% CI +8.4 to +13.4; *p* < 0.001); and uPDI +5.4 (95% CI +3.4 to +7.4; *p* < 0.001). The change in all three scores significantly correlated with change in body weight, with PDI (r = −0.40; *p* < 0.001) and hPDI (r = −0.37; *p* < 0.001) having stronger correlations than uPDI (r = −0.21; *p* = 0.002).

### PDI food scores

The scores for fruits, vegetables, whole grains, and legumes increased significantly in the vegan group (Table 1), and changes were negatively associated with changes in body weight (Table 2). The consumption of all animal products decreased in the vegan group. Changes were positively associated with changes in body weight. The change in vegetable oil intake correlated positively with weight changes (r = +0.27; *p* < 0.001). A multiple linear regression model (r^2^ = 0.26; *p* < 0.001) showed that the following food components were independently associated with weight loss: whole grains (*p* = 0.006) and legumes (*p* = 0.007) showed a negative association, while meat (*p* < 0.001), vegetable oils (*p* = 0.006), and sweets (*p* = 0.01) showed a positive association. The R-squared is about 0.26 for this model.

**Table 1:** Plant-based dietary index (PDI), healthful (hPDI), and unhealthful plant-based dietary index (uPDI), and the individual food components at baseline and 16 weeks.

	Vegan group			Control group			Effect size	*P* value
	Week 0	Week 16	Change	Week 0	Week 16	Change		
PDI	52.9 (51.5–54.3)	62.3 (61.3–63.3)	+9.4 (+7.8 to +10.9)***	52.8 (51.5–54.0)	51.6 (50.0–53.1)	−1.2 (−2.6 to +0.1)	+10.6 (+8.6 to +12.6)	*p* < 0.001
hPDI	55.4 (53.7–57.1)	67.7 (66.8–68.6)	+12.3 (+10.6 to +14.0)***	55.8 (54.0–57.7)	57.2 (55.3–59.1)	+1.4 (−0.4 to +3.2)	+10.9 (+8.4 to +13.4)	*p* < 0.001
uPDI	53.8 (52.4–55.1)	59.0 (58.0–60.0)	+5.2 (+3.8 to +6.6)***	53.5 (52.1–54.9)	53.3 (51.7–54.8)	−0.2 (−1.6 to +1.2)	+5.4 (+3.4 to +7.4)	*p* < 0.001
PDI Food Components (points)								
Fruits	3.0 (2.7–3.2)	3.8 (3.6–4.0)	+0.8 (+0.5 to +1.1)***	3.0 (2.7–3.3)	3.0 (2.7–3.2)	−0.0 (−0.4 to +0.3)	+0.8 (+0.4 to +1.3)	*p* < 0.001
Vegetables	3.0 (2.7–3.3)	3.8 (3.5–4.0)	+0.8 (+0.5 to +1.1)***	3.0 (2.7–3.3)	3.0 (2.7–3.3)	+0.0 (−0.3 to +0.4)	+0.8(+0.3 to +1.2)	0.001
Whole Grains	3.0 (2.7–3.2)	4.1 (3.9–4.4)	+1.2 (+0.8 to +1.5)***	3.0 (2.7–3.3)	2.7 (2.4–2.9)	−0.3 (−0.0 to–0.7)	+1.5 (+1.0 to +1.9)	*p* < 0.001
Nuts	2.8 (1.4–2.6)	2.1 (1.0–1.9)	−0.8 (−1.0 to +1.5)***	3.1 (2.8–3.4)	3.0 (2.7–3.3)	−0.1(−0.4 to +0.2)	−0.6 (−0.2 to −1.1)	0.003
Legumes	2.7 (1.6–2.4)	4.3 (4.0–4.5)	+1.6 (+1.2 to +1.9)***	2.8 (2.4–3.1)	2.6 (2.3–2.9)	−0.2 (−0.5 to +0.2)	+1.7 (+1.3 to +2.2)	*p* < 0.001
Vegetable Oils	3.2 (2.9–3.4)	1.9 (1.7–2.1)	−1.3 (−1.6 to +1.9)***	2.8 (2.5–3.1)	2.9 (1.5–2.6)	+0.1 (−0.2 to +0.5)	−1.4 (−0.9 to −1.9)	*p* < 0.001
Coffee and Tea	2.9 (2.7–3.2)	2.8 (2.5–3.0)	−0.2 (−0.4 to +0.0)	3.0 (2.8–3.3)	3.0 (2.7–3.3)	−0.0 (−0.3 to +0.2)	−0.2 (−0.5 to +0.2)	0.34
Fruit Juice	2.6 (2.3–2.9)	3.0 (2.7–3.3)	0.4 (−0.0 to +0.8)	2.8 (2.4–3.1)	2.5 (2.2–2.9)	−0.2 (−0.6 to +0.2)	+0.6 (+0.1 to +1.2)	0.03
Sugar Sweetened Beverages	2.2 (1.9–2.5)	1.7 (1.5–2.0)	−0.5 (−0.9 to–0.2)**	2.6 (2.3–3.0)	2.4 (1.8–2.1)	−0.2 (−0.5 to +0.1)	−0.7 (−0.3 to −1.1)	0.20
Refined Grains	3.1 (2.9–3.4)	1.9 (1.7–2.1)	−1.3 (−1.6 to–0.9)***	2.8 (2.6–3.1)	2.0 (1.8–2.2)	−0.8 (−1.2 to–0.5)	−0.4 (−0.9 to +0.0)	0.07
Potatoes	2.7 (2.3–3.0)	2.7 (2.3–3.0)	+0.0 (−0.4 to +0.4)	2.5 (2.2–2.8)	2.6 (2.3–2.9)	+0.1 (−0.33 to +0.5)	−0.1 (−0.7 to +0.5)	0.74
Sweets	3.2 (3.0–3.5)	3.1(2.8–3.3)	−0.1 (−0.5 to +0.2)	2.8 (2.5–3.0)	2.6 (2.3–2.9)	−0.2 (−0.5 to +0.2)	+0.4 (+0.0 to +0.8)	0.87
Animal Fats	3.0 (2.7–3.3)	4.5 (4.4–4.7)	+1.6 (+1.3 to +1.9)	3.1 (2.8–3.4)	3.3 (3.0–3.7)	+0.2(−0.2 to +0.6)	+1.3 (+0.9 to +1.9)	*p* < 0.001
Dairy	3.1(2.8–3.3)	5.0 (4.9–5.0)	+1.9 (+1.6 to +2.2)***	2.9 (2.7–3.2)	3.1 (2.8–3.4)	+0.2(−0.1 to +0.5)	+1.7 (+1.3 to +2.1)	*p* < 0.001
Eggs	3.1 (2.8–3.4)	4.9 (4.9–5.0)	+1.8 (+ 1.6 to +2.1)***	3.2 (2.9–3.5)	3.2 (2.9–3.5)	+0.0 (−0.3 to +0.3)	+1.8 (+1.4 to +2.2)	*p* < 0.001
Animal Misc.	3.0	3.0	+0.0	3.0	3.0	+0.0	0 (0 to 0)	-
Meat	3.0 (2.8–3.3)	5.0 (5.0–5.0)	+1.9 (+1.7 to +2.2)***	3.0 (2.7–3.2)	3.1 (2.8–3.4)	+0.2 (−0.1 to +0.4)	+1.8 (+1.4 to +2.2)	*p* < 0.001
Seafood	3.4 (3.1–3.7)	4.9 (4.8–5.0)	+1.5 (+1.2 to +1.8)***	3.4 (3.0–3.7)	3.4 (3.1–3.8)	+0.1 (−0.3 to +0.5)	+1.5 (+1.0 to +2.0)	*p* < 0.001
TOTAL	53.0 (51.5–54.0)	62.3 (61.3–63.3)	+9.4 (+7.8 to +11.0)***	52.8 (51.5–54.0)	51.6 (50.0–53.1)	−1.2 (−2.6 to +0.1)	+10.6 (+8.6 to +12.6)	*p* < 0.001

Data are presented as means with 95% confidence intervals.

*p < 0.05; **p < 0.01; ***p < 0.001.

**Table 2:** Pearson correlations between changes in body weight and changes in the individual food components of the plant-based dietary index (PDI).

PDI Food Components	Δ Body weight
Fruits	r = −0.20; *p* = 0.003
Vegetables	r = −0.19; *p* = 0.005
Whole Grains	r = −0.33; *p* < 0.001
Nuts	r = +0.09; *p* = 0.18
Legumes	r = −0.29; *p* < 0.001
Vegetable Oils	r = +0.27; *p* < 0.001
Coffee and Tea	r = +0.03; *p* = 0.63
Fruit Juice	r = −0.01; *p* = 0.83
Sugar Sweetened Beverages	r = +0.07; *p* = 0.33
Refined Grains	r = +0.10; *p* = 0.14
Potatoes	r = −0.08; *p* = 0.26
Sweets	r = +0.09; *p* = 0.16
Animal Fats	r = +0.19; *p* = 0.005
Dairy	r = +0.20; *p* = 0.003
Eggs	r = +0.21; *p* = 0.002
Meat	r = +0.36; *p* < 0.001
Seafood	r = +0.18; *p* = 0.008

After Bonferroni correction, *p* values less than 0.003 (0.05/17) are considered significant.

## Discussion

In a 2021 study in three U.S. prospective cohorts, increased PDI and hPDI were associated with a lower risk of type 2 diabetes; changes in uPDI were not [4]. These findings were confirmed by an analysis of their plasma metabolite profiles [5]. Our findings show, however, that changes in all plant-based indices—PDI, hPDI, and uPDI were inversely associated with weight changes. Replacing animal products with either so-called healthful or unhealthful plant-based foods was associated with weight loss, and change in oil intake was positively associated with change in body weight. The food components that were independently associated with weight loss included whole grains and legumes, which showed a negative association, while meat, vegetable oil, and sweets showed a positive association.

The strengths of the current trial include a randomized, parallel design, which accounted for seasonal effects. The study also has limitations. The PDI scores were based on self-reported diet records. The participants were volunteers and may not represent the general population.

In conclusion, all three scores increased in the vegan group and correlated negatively with changes in body weight. These findings suggest that minimizing the consumption of animal products and vegetable oil may be an effective weight-loss strategy.

### Supplementary information

Supplement 1

Supplementary Figure 1
