# Supplementary figures and images for: Does diet quality matter? A secondary analysis of a randomized clinical trial
Source: Eur J Clin Nutr. 2023 Nov 28;78(3):270–3. doi: 10.1038/s41430-023-01371-y (PMC10927534; doi:10.1038/s41430-023-01371-y)

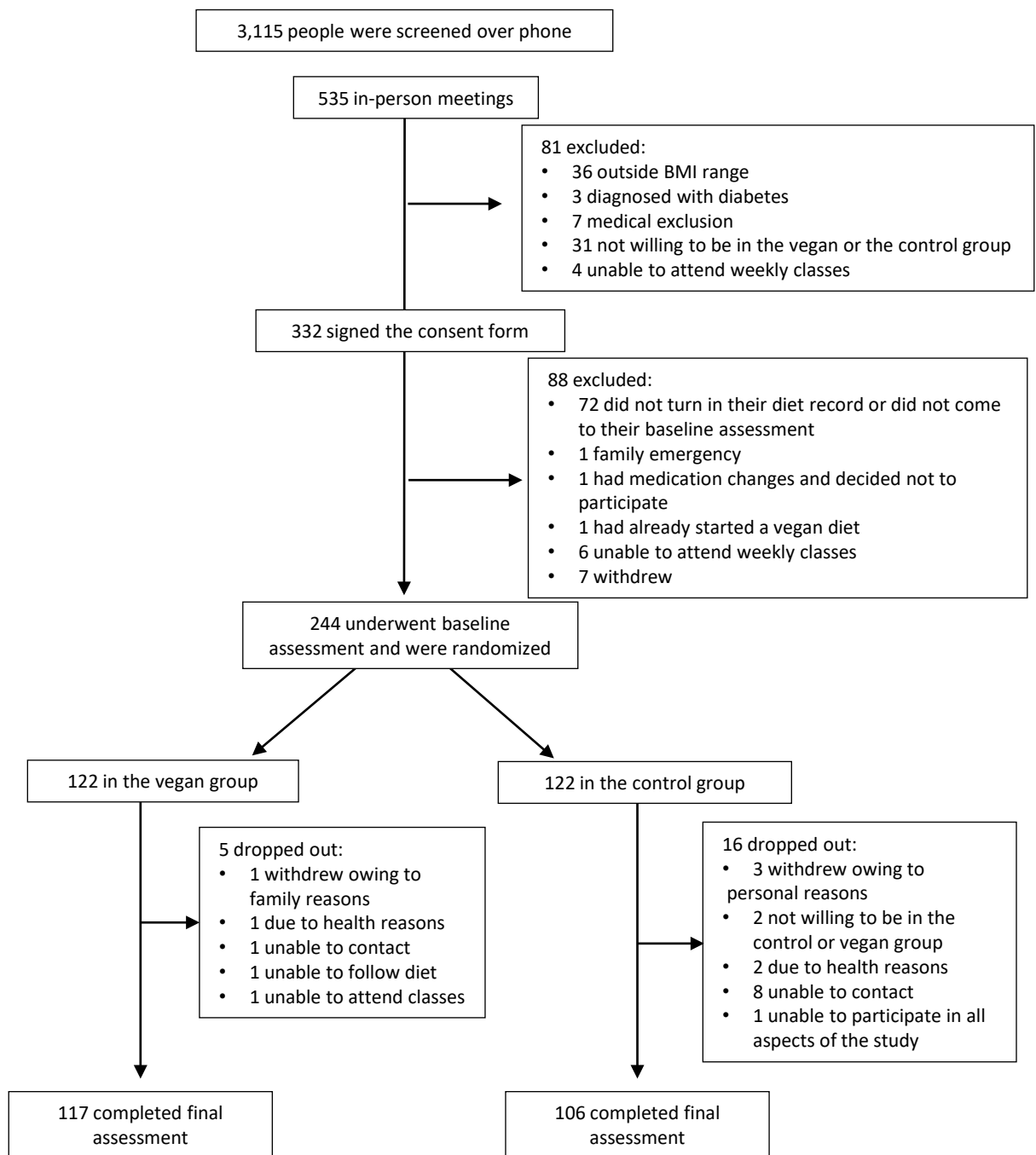

**Suppl. Fig. 1. Participant Flow Chart.**

Supplement: Supplementary file 2 — Supplementary Figure 1 [file 41430_2023_1371_MOESM2_ESM.pdf]
